# Site-Independent Hydrogenation Reactions on Oxide-Supported Au Nanoparticles Facilitated by Intraparticle Hydrogen Atom Diffusion

**DOI:** 10.1021/acscatal.1c01987

**Published:** 2021-07-21

**Authors:** Shahar Dery, Hillel Mehlman, Lillian Hale, Mazal Carmiel-Kostan, Reut Yemini, Tzipora Ben-Tzvi, Malachi Noked, F. Dean Toste, Elad Gross

**Affiliations:** † Institute of Chemistry, The Hebrew University, Jerusalem 91904, Israel; ‡ The Center for Nanoscience and Nanotechnology, The Hebrew University, Jerusalem 91904, Israel; § Department of Chemistry, University of California, Berkeley, California 94720, United States; ∥ Department of Chemistry, Bar Ilan University, Ramat Gan 5290002, Israel; ⊥ Bar-Ilan Institute of Nanotechnology and Advanced Materials, Ramat Gan 5290002, Israel

**Keywords:** metal−support interactions, IR nanospectroscopy, Au nanoparticles, metal oxide, hydrogenation, single-particle measurements

## Abstract

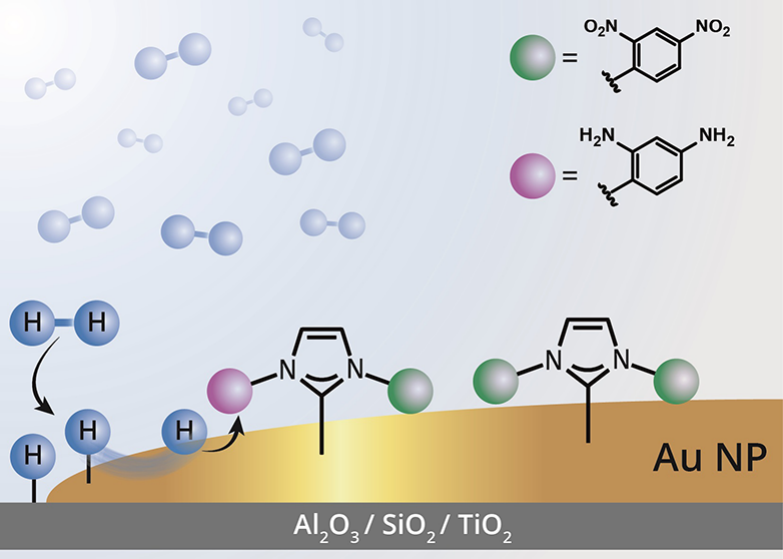

Metal–support interactions have been widely utilized for optimizing the catalytic reactivity of oxide-supported Au nanoparticles. Optimized reactivity was mainly detected with small (1–5 nm) oxide-supported Au nanoparticles and correlated to highly reactive sites at the oxide–metal interface. However, catalytically active sites are not necessarily restricted to the interface but reside as well on the Au surface. Uncovering the interconnection between reactive sites located at the interface and those situated at the metal surface is of crucial importance for understanding the reaction mechanism on Au nanoparticles. Herein, high-spatial-resolution IR nanospectroscopy measurements were conducted to map the localized reactivity in hydrogenation reactions on oxide-supported Au particles while using nitro-functionalized ligands as probes molecules. Comparative analysis of the reactivity pattern on single particles supported on various oxides revealed that oxide-dependent reactivity enhancement was not limited to the oxide–metal interface but was detected throughout the Au particle, leading to site-independent reactivity. These results indicate that reactive Au sites on both the oxide–metal interface and metal surface can activate the nitro groups toward hydrogenation reactions. The observed influence of oxide support (TiO_2_ > SiO_2_ > Al_2_O_3_) on the overall reactivity pattern specified that hydrogen dissociation occurred at the oxide–metal interface, followed by highly efficient intraparticle hydrogen atom diffusion to the interior parts of the Au particle. In contrast to Au particles, the oxide–metal interface had only a minor impact on the reactivity of supported Pt particles in which hydrogen dissociation and nitro group reduction were effectively activated on Pt sites. Single-particle measurements provided insights into the relative reactivity pattern of oxide-supported Au particles, revealing that the less-reactive Au metal sites can activate hydrogenation reactions in the presence of hydrogen atoms that diffuse from the Au/oxide boundary.

## Introduction

Hydrogenation of unsaturated bonds in organic molecules by solid catalysts is a vital process in the chemical industry.^1,2^ The activation mechanism of hydrogenation reactions can be divided to two separate steps: (i) dissociation of hydrogen molecules into adsorbed hydrogen atoms and (ii) chemisorption of the unsaturated precursor, which involves a double-bond rehybridization to afford the sequential hydrogenation.^3,4^

**Figure 1:**
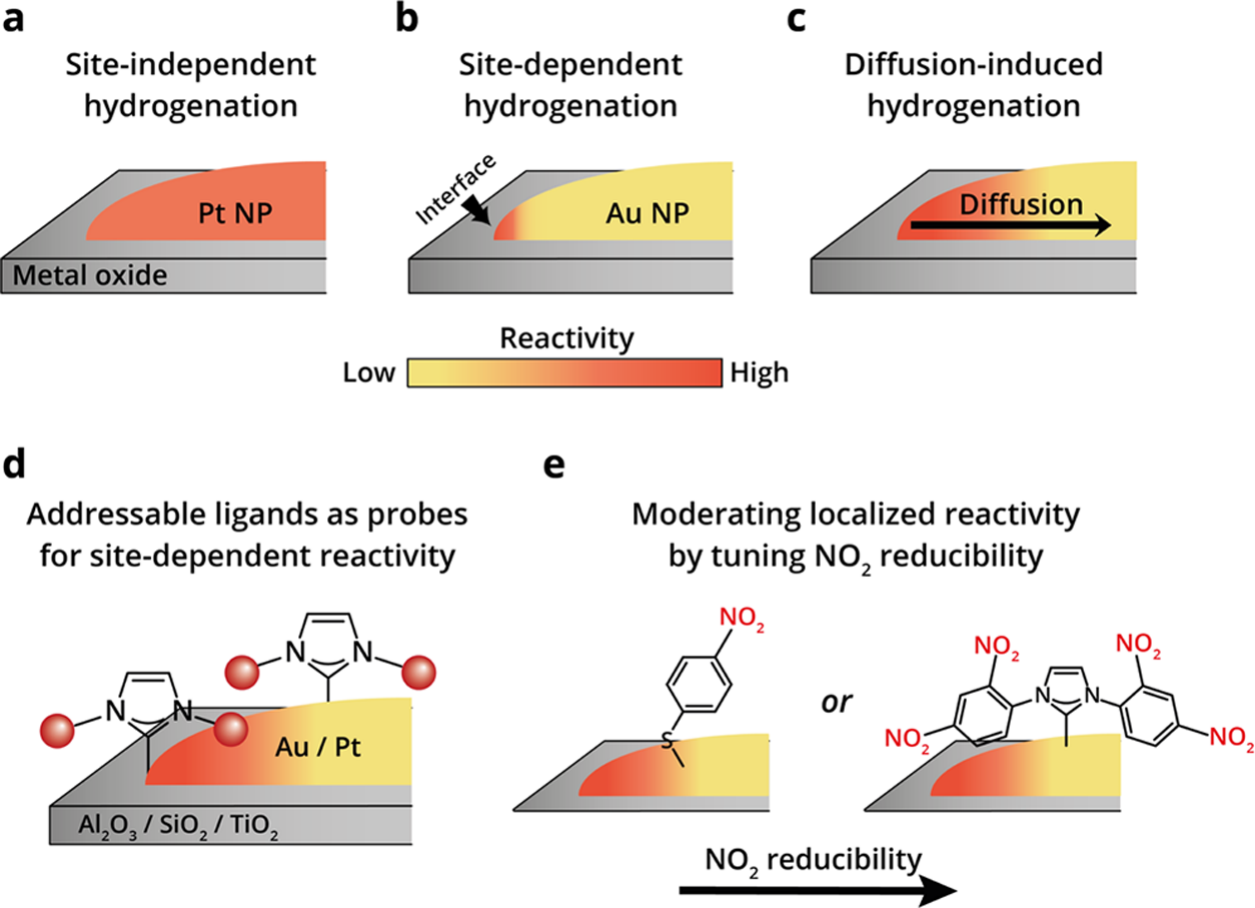
Schematic description of hydrogenation reaction on oxide-supported nanoparticles and the experimental setup for mapping site-dependent hydrogenation. Two main scenarios for hydrogenation were previously identified: H_2_-dissociative chemisorption and unsaturated bond activation are homogeneously activated on the metal surface (a) or locally confined to the oxide–metal interface (b). In this work, a third scenario (c) was identified in which unsaturated bond activation was facilitated across the surface of the particle, wherein H_2_-dissociative chemisorption was activated at the oxide–metal interface. Highly efficient intraparticle hydrogen atom diffusion from the interface led to site-independent hydrogenation reaction. (d) To map the local reactivity of oxide-supported nanoparticles, addressable ligands were used as probes and their chemical signature was characterized by IR nanospectroscopy measurements. (e) Reactivity variations on surface sites were analyzed using nitro-functionalized *N*-heterocyclic carbenes (NHCs) and thiols that differ in their reducibility.

On highly reactive metals, such as Pt, these two steps are facilitated throughout the metal surface to exhibit site-independent hydrogenation reactivity (Figure 1a).^5−7^ Hydrogenation reactions on less-reactive metals, such as Au, requires deposition of the metal nanoparticles on reducible metal–oxide supports.^3,8−10^ These catalytic systems are characterized with high and localized reactivity at the oxide–metal interface (Figure 1b).^10−12^ Reactivity enhancement at the interface was attributed to high density of low-coordinated metal sites^13−16^ or local modification of the electronic properties of the metal atoms at the interface.^17−25^

However, catalytically active sites may not only appear at the metal–oxide interface but can also reside across the metal surface. Therefore, a third scenario should also be taken into account in which the hydrogenation reaction is activated on two different sites—H_2_ dissociation occurs on one site and double-bond activation and hydrogenation is facilitated on a remote site (Figure 1c).^26−30^ In this scenario, the hydrogen atom needs to travel from the highly reactive sites at the metal–oxide interface, in which dissociative chemisorption of H_2_ is activated, to the interior part of the particle. Since hydrogen atom diffusion on metal surfaces is a rapid process,^31−33^ the hydrogenation yield in the interior part of the particle will mostly depend upon the ability of metal atoms to strongly interact with the unsaturated group in the reactant molecule.^34,35^

The efficiency of inter- and intraparticle diffusion of reactive species,^29,30^ such as hydrogen atoms, was widely documented.^11,36−38^ Thus, it is expected that the limiting step for hydrogenation reactions on sites that are remote from the metal–oxide interface will be dominated by the interaction between the unsaturated group and interior metal surface sites. As a result, site-dependent reactivity analysis will be essential to clearly identify the catalytic role of sites that are located at the oxide–metal interface versus sites that are located in the interior part of the particle.^39^ Analysis of the reactivity on these metal sites is at the core of this study.

To identify site-dependent hydrogenation reaction, we developed a model system constructed of self-assembled monolayers (SAMs) that were functionalized with reducible nitro groups (Figure 1d). The chemically addressable SAMs decorated the surface of Au and Pt particles, which were deposited on TiO_2_, SiO_2_, or Al_2_O_3_ films, which differ in their reducibility.^40−42^ Using chemically addressable SAMs as an indicator of surface-induced reactivity, we were able to directly compare the influence of distinct surface sites (interface vs interior) toward nitro groups hydrogenation. The reactivity on different sites of oxide-supported nanoparticles was documented by conducting high-spatial-resolution IR nanospectroscopy measurements.^13,43−45^*N*-heterocyclic carbenes (NHCs) and thiols, two distinct classes of surface ligands that differ in their tendency toward nitro reduction, were used to meticulously moderate the interaction between the reducible group and the catalytic surface (Figure 1e).

High-spatial-resolution IR measurements revealed site-independent reactivity on all oxide-supported Au particles. These results indicated that metal sites that are located at the interior part of the Au particle can effectively activate the nitro bond toward reduction in a similar yield to sites that are located at the metal–oxide interface. However, interface sites were essential for molecular hydrogen dissociation in which it was probed that TiO_2_ > SiO_2_ > Al_2_O_3_ in activating the dissociation reaction. In contrast to Au nanoparticles, in which hydrogen dissociation and double-bond activation occur on different sites, on highly reactive Pt particles, both steps were uniformly facilitated throughout the metal surface with almost no impact of the metal–oxide interface on reactivity.

## Experimental Section

### Sample Preparation

Si(110) wafer (2 cm × 2 cm) was cleaned with Piranha and subsequently coated with an oxide layer by atomic layer deposition (ALD) (Ultratech Savannah 200). Al_2_O_3_ films were prepared under the following conditions: the chamber was preheated to 200 °C and obtained under 0.12 Torr of argon flow. The aluminum precursor (TMA—trimethyl aluminum, Sigma-Aldrich) and the water used as oxygen precursor were held at room temperature. Each ALD cycle consisted of 0.02 s exposure to H_2_O vapor followed by 12 s argon purge, and then 0.02 s exposure to TMA followed by 12 s of argon purge. The entire procedure was repeated for 200 cycles. TiO_2_ films were prepared under the following conditions: the chamber was preheated to 150 °C and obtained under 0.12 Torr of argon flow. The titanium precursor (TDMAT—tetrakis(dimethylamido)titanium(IV), Sigma-Aldrich) was preheated to 75 °C, and water (at room temperature) used as oxygen precursor. Each ALD cycle consisted of 0.015 s exposure to H_2_O vapor followed by 5 s argon purge, and then 0.05 s exposure to TDMAT followed by 5 s of argon purge. The procedure was repeated for 425 cycles. Native SiO_2_/Si(110) was used after rinsing with ethanol.

Metallic particles were prepared by thermal evaporation of Pt or Au (15 nm layer thickness at a deposition rate of 0.3 nm/s) on an oxide-coated Si wafer. This process was followed by annealing to 550 °C for 5 h under 1 atm N_2_, leading to particles’ formation in the size range of 150 ± 50 nm. Cross-sectional and profile analysis of the particles was conducted by atomic force microscopy (AFM) measurements. The particles’ height was 50 ± 5 nm and their profile length was 20 ± 4 nm. This preparation method does not involve the addition of any organic molecules, thus assuring that all IR signals originate from the surface-anchored probe molecules and not from organic residues. The inhomogeneity in particles’ size and shape, which is induced by this preparation method, enables to probe if any size- or structure-dependent reactivity exists within various particles. Surface anchoring of *para*-nitrothiophenol (*p*-NTP) was performed by immersing the sample in 5 mM *p*-NTP in ethanol for 12 h. NO_2_-NHC deposition was performed based on published protocols.^43^

### Synchrotron Infrared Nanospectroscopy (SINS) Measurements

Synchrotron infrared light (provided by the Advanced Light Source (ALS), Lawrence Berkeley National Laboratory) was focused onto the apex of a metal-coated AFM tip (NCH-Pt by Nanosensors or nano-FTIR probes by Neaspec) in an AFM system (Innova, Bruker at beamline 5.4 or a neaSNOM, Neaspec at beamline 2.4). As the near-field scattered signal depends nonlinearly on the distance between the tip and the sample, the tip oscillation induces higher harmonics (nω) in the near-field scattered signal. Consequently, the near-field signal was differentiated from the far-field background by detecting the high harmonics frequency of 2ω with a lock-in amplifier. Following AFM topography imaging of the surface, infrared nanospectroscopy point measurements were conducted at selected locations. The Fourier transform of the interferogram provides a complex-valued near-field spectrum. The real (Re_(ν)_, where ν is the wavenumber) and imaginary (Im_(ν)_) spectra can be represented as spectral amplitude (*A*_(ν)_) and phase (ϕ_(ν)_). The near-field spectra were reported in the form of a normalized scattering phase (ϕ_(ν)_ = ϕ_sample(ν)_ – ϕ_reference(ν)_), using the Si surface as a reference point. IR nanospectroscopy measurements were conducted on different sites on the surface of at least 10 particles under each reaction conditions in every sample. Prior to each acquisition of IR spectrum, the sample reached a thermal equilibrium with thermal drift level of <5 nm/min. Since the acquisition duration was 3 min, the sample drift during IR measurement was <15 nm. The IR signal was continuously monitored during acquisition to assess the tip position and measurement location.

### Near-Ambient-Pressure X-ray Photoelectron Spectroscopy (NAP-XPS)

Measurements were performed at beamline 11.0.2 of the Advanced Light Source (ALS) at Lawrence Berkeley National Laboratory using APXPS-1 endstation. XPS spectra of C 1s, N 1s, Pt 4f, and Au 4f were acquired with a beam energy of 525.0 eV. NAP-XPS measurements were performed at variable temperature and 0.1 Torr H_2_. The binding energies were calibrated according to the core level (4f_7/2_) position of Au and Pt, located at 84.0 and 71.2 eV, respectively. Data analysis was performed using CasaXPS software.

## Results and Discussion

**Figure 2:**
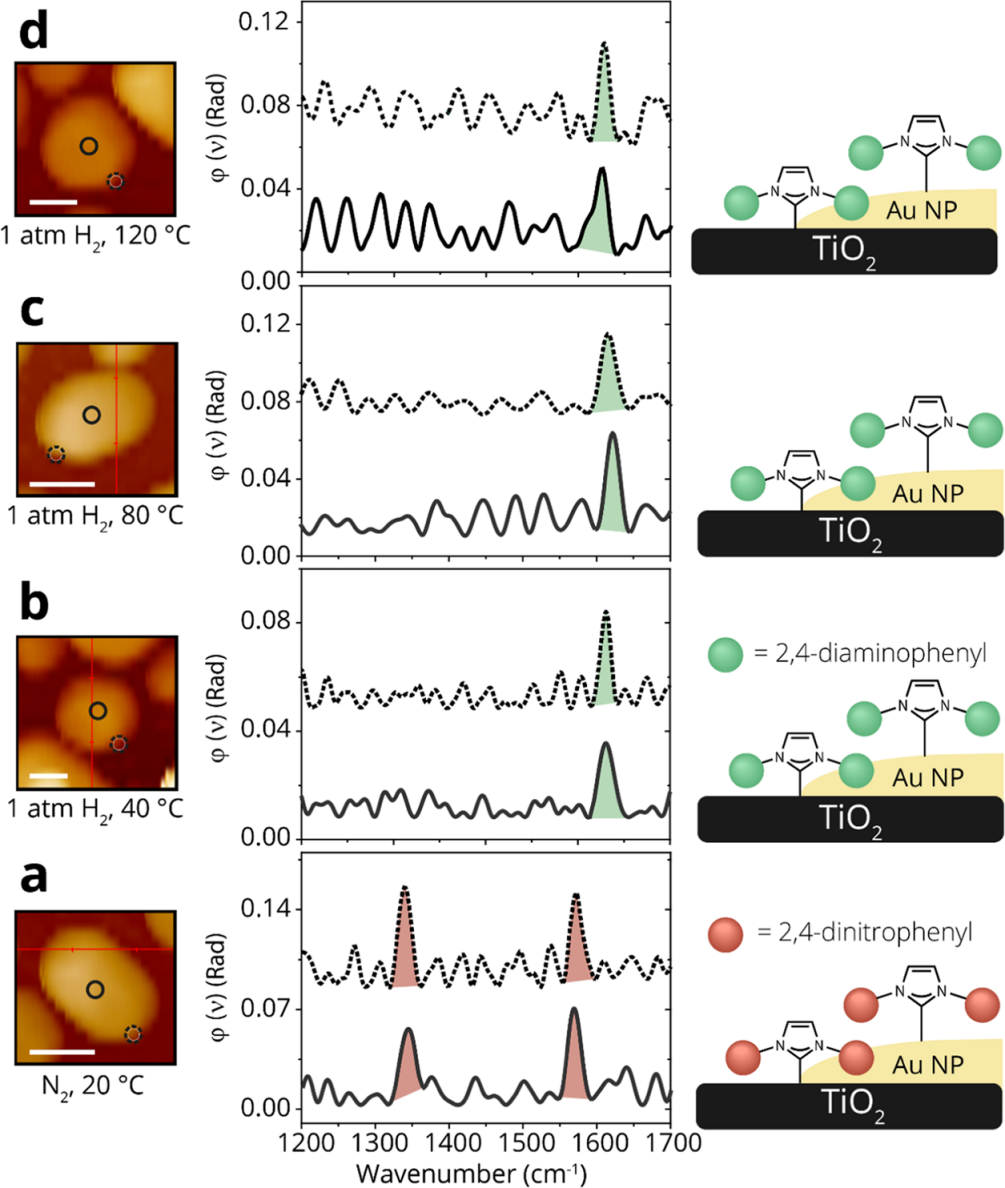
AFM images and IR nanospectroscopy measurements of TiO_2_-supported Au particles that were coated with NO_2_-NHCs and exposed to various reducing conditions. The topography of Au particles was measured by AFM (left). IR spectrum was locally measured at the center and edge of the probed particles (middle). The location of the IR measurement is marked by solid and dashed circles in the AFM image, and the corresponding IR spectra are plotted in solid and dashed lines. Schematic description of the NHCs located on the center and edge of the Au particle, as identified by local IR measurements (right). AFM and IR nanospectroscopy measurements were conducted at rt (a) and after exposure of the sample to 1 atm H_2_ at 40 (b), 80 (c), and 120 °C (d). The IR peaks were colored for clarity. Nitro- and amine-related peaks were colored in red and green, respectively. Scale bar in all AFM images is 100 nm.

Au particles of size 150 ± 50 nm were prepared by thermal annealing (550 °C, 1 atm N_2_, 5 h) of a 15 nm thick Au film that was deposited on TiO_2_, Al_2_O_3_, or SiO_2_ films (Supporting Information Figures S1–S3). The large size of the probed particles (>100 nm) along with low thermal drift (<5 nm/min) ensured that reactivity variations between interface sites and sites that are located in the interior part of the particle will be probed using IR nanospectroscopy measurements, characterized with a spatial resolution of 20 nm. Ensuing particles’ formation, the metal surface was coated with a monolayer of nitro-functionalized NHCs (NO_2_-NHCs). The surface-anchored NO_2_-NHCs function as molecular probes for hydrogenation reactions. The topography of oxide-supported Au particles was mapped by atomic force microscopy (AFM) measurements and the vibrational spectrum on different sites of the NO_2_-NHCs coated Au particles was measured by IR nanospectroscopy (Figure 2).

An AFM image of TiO_2_-supported Au particles that were coated with NO_2_-NHCs is shown in Figure 2a (left). Since Au/TiO_2_ interface is well known for its efficient hydrogen activation,^12,23^ this system provides an excellent experimental setting to identify site-dependent reactivity variations. IR nanospectroscopy measurements were conducted at the center and edge of the probed particle. The locations of the IR measurements were marked in the AFM image by full and dashed circles. The corresponding IR spectra are shown in Figure 2a (middle), using full and dashed lines for indication of the spectra that were collected at the center and edge of the particle, respectively. Two dominant peaks, located at 1330 and 1570 cm^–1^ and assigned to symmetric and asymmetric nitro stretches, respectively, were identified in the IR spectra that were measured on the edge and central part of Au particle. The similarity in the IR spectra that were measured on both sites indicated that the nitro-functionalized species were homogeneously distributed on the metal surface and were not reduced under ambient conditions.

In this stage, hydrogenation was initiated by exposure of the sample to mild reducing conditions (1 atm H_2_, 40 °C, 10 h), which was followed by IR nanospectroscopy measurements. Notably, the nitro-correlated IR peaks were not observed after exposure of the sample to mild reducing conditions, while a new peak at 1625 cm^–1^ was detected and attributed to N–H bending of a primary amine (Figure 2b). No differences were identified between the IR spectrum that was measured on the side and center of the Au particle, indicating that nitro-to-amine reduction was conducted on both sites. Exposure of the sample to harsher reducing conditions, in which the reduction temperature was elevated to 80 and 120 °C, did not change the acquired IR signals as shown in Figure 2c,d, respectively. Comparable reactivity was measured on the edge and central part of the particles under all reducing conditions, with no deformation or noticeable desorption of NO_2_-NHCs from the Au surface. Therefore, the spectroscopic data demonstrates that the nitro groups in NO_2_-NHCs were fully reduced following exposure of TiO_2_-supported Au particles to 1 atm H_2_ at 40 °C.

**Figure 3:**
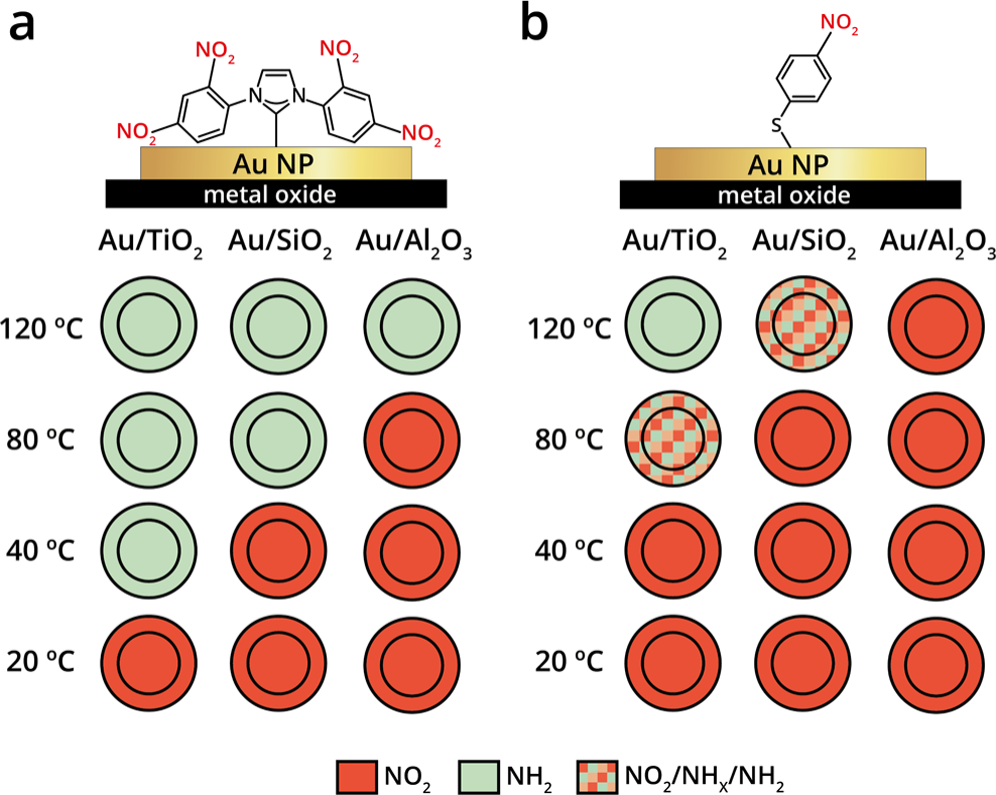
Schematic representation of the averaged reactivity pattern toward nitro reduction of NO_2_-NHCs and *p*-NTP that were deposited on oxide-supported Au particles. The reactivity toward nitro reduction of (a) NO_2_-NHC- and (b) *p*-NTP-coated Au particles that were deposited on different oxides was probed by IR nanospectroscopy measurements and the spectroscopic results are schematically shown. The outer ring and the inner circle correspond to the reactivity that was detected at the oxide–metal interface and on the central part of the particles, respectively. The reactivity on the different sites was color-coded. The nitro group was color-coded in red, and the amine group was color-coded in green.

A similar pattern of site-independent reactivity toward nitro reduction was measured on several TiO_2_-supported Au particles (up to 10 particles were measured for each reducing condition) at various sizes on different regions of the sample (Supporting Information Figure S4). The averaged reactivity pattern toward nitro reduction of Au/TiO_2_, as identified by IR nanospectroscopy measurements, is schematically illustrated in Figure 3a (left column). In this scheme, the probed Au particles are illustrated as a circle, in which the outer ring represents the reactivity that was measured at the oxide–metal interface, while the inner circle corresponds to the reactivity that was measured at the inner part of the particle. The colors in the inner circle and outer ring represent the detection of either NO_2_- or NH_2_-functionalized NHCs, which are respectively colored in red or green. NO_2_-NHCs were detected across the TiO_2_-supported Au particles at 20 °C, as depicted by the red-colored inner and outer circles. Exposure of the particles to reducing conditions (1 atm H_2_, 40 °C) led to nitro-to-amine reduction throughout the surface of the particle, as shown by the green-colored inner and outer circles.

It was previously demonstrated that dissociative chemisorption of hydrogen is facilitated on the highly reactive Au/TiO_2_ interface and not on a flat Au surface.^10,11^ Thus, the uniform reactivity toward nitro reduction across the surface of TiO_2_-supported Au particle indicates that: (i) effective surface diffusion of hydrogen atoms from the interface to the center of the Au particle has occurred; (ii) Au sites that are remote from the interface can interact with the nitro group strongly enough to enable reduction. Therefore, the site-independent reactivity indicates that both hydrogen atom diffusion and nitro group activation are effectively activated on large (>100 nm) Au nanoparticles.

To moderate the effect of the oxide support on reactivity, Au nanoparticles were deposited on less-reducible oxides to decrease the dissociative chemisorption of hydrogen molecules at the interface. The nitro reduction reaction of NO_2_-NHCs was studied on Au particles that were supported on SiO_2_ and Al_2_O_3_, which are less reducible in comparison to TiO_2_.^40,42^

IR nanospectroscopy measurements of NO_2_-NHC/Au/SiO_2_ and NO_2_-NHC/Au/Al_2_O_3_ are shown in Supporting Information Figures S5 and S6, respectively. Schematic representation of the averaged reactivity pattern, based on IR nanospectroscopy measurements of ∼10 different nanoparticles for each support, is presented in Figure 3a. Nitro-to-amine reduction was detected at a reduction temperature of 80 and 120 °C for SiO_2_- and Al_2_O_3_-supported Au particles, respectively. In both cases, there were no noticeable differences between the IR spectra that were acquired at the oxide–metal interface and the center of the particle.

The signal-to-noise ratio in each IR spectrum was between 2:1 and 4:1. The dominant peaks in the spectrum were the symmetric and asymmetric nitro vibrations and N–H bending, which were detected on different sites and on different particles with relatively small variations (±7 cm^–1^) in their peak position. An additional feature was detected at 1200–1300 cm^–1^ in some of the measurements performed in proximity to the metal/oxide interface. This feature was attributed to SiO_2_ sites that were partially exposed at the interface (Supporting Information Figure S7).^46^ All other features were close to the noise level and were characterized with random distribution. The changes in the signal-to-noise ratio on different sites can result from local variations in the particle’s structure, which can impact the IR nanospectroscopy signal due to its surface-dependent signal enhancement mechanism.^44^

Analysis of the reactivity pattern of oxide-supported Au particles toward NO_2_-NHCs reduction unveiled two key conclusions:

(i). The oxide support tuned the reactivity in the following order TiO_2_>SiO_2_>Al_2_O_3_, thus certifying the critical role of the oxide–metal interface in activating the dissociative chemisorption of hydrogen.^47,48^ The superior reactivity of TiO_2_-supported Au particles was correlated to charge transfer between the metal and the support, as probed by X-ray photoelectron spectroscopy (XPS) measurements. The Au4f_7/2_ XPS peak position in Au/TiO_2_ was shifted by 0.4 eV, in comparison to that of Au/SiO_2_ and Au/Al_2_O_3_ (Supporting Information Figure S8), indicative of charge transfer from the metal to the support.^49−51^ The enhanced activity of SiO_2_ compared with Al_2_O_3_ was attributed to the stronger Lewis acidity of the SiO_2_ support.^52,53^
(ii). IR nanospectroscopy measurements revealed site-independent reactivity of Au particles on the three different oxides. These results show that in all measured samples, surface diffusion of hydrogen atoms from the oxide–metal interface effectively occurred and led to homogeneous reactivity across the particle surface. Although dissociative chemisorption of hydrogen was initiated at different temperatures as a function of the oxide’s properties, it constituted a rate-limiting step for nitro reduction in all three interfaces. Indications for sequential hydrogenation steps on different surface sites were not observed on the Au particle; therefore, these steps were presumed to have lower activation energy barriers.

The extended reaction duration (10 h) and the use of a monolayer as a model reactant ensured that nitro reduction will not be kinetically limited by atomic hydrogen coverage. It should be noted that elevated temperature (>40 °C) was essential for nitro reduction catalyzed by Au supported on Al_2_O_3_ or SiO_2_, demonstrating the crucial role of Au/oxide interface in activation of hydrogen dissociation. The oxide influence on the reactivity of Au nanoparticles in hydrogenation reaction, in which TiO_2_ > SiO_2_ > Al_2_O_3_ was also identified in crotonaldehyde hydrogenation.^54^

The exceptional reactivity of small (3 nm) TiO_2_-supported Au nanoparticles toward hydrogenation reactions,^55^ and specifically nitroaromatics reduction,^56,57^ which was previously reported, is connected with the ability to provide sufficient hydrogen atoms to surface Au atoms. In contrast, lower reaction rates were detected on larger nanoparticles, which can be attributed to kinetic limitations resulting from reduced hydrogen coverage on the surface.

The reducibility of dinitrophenyl moieties results in the high reactivity toward nitro reduction of NO_2_-NHCs.^47,58,59^ To further examine the generality of site-independent reduction in oxide-supported metal particles, the localized reactivity was probed using *para*-nitrothiophenol (*p*-NTP) as a chemical marker. The feasibility for using nitrogen-based thiols as markers for probing reactions on metallic surfaces was previously demonstrated by tip-enhanced Raman spectroscopy (TERS) measurements.^60−63^

Unlike surface-anchored NO_2_-NHCs, in which the dinitrophenyl groups reside in proximity to the metal surface, the *p*-NTP molecules self-assemble in a close to vertical adsorption geometry.^64^ These differences in the anchoring geometry combined with the fact that *p*-NTP bears a nitrophenyl ring, compared to dinitrophenyl in NO_2_-NHCs, make *p*-NTPs less susceptible for hydrogenation. Accordingly, the use of *p*-NTP as probe molecule for site-dependent reactivity analysis allows moderating of the hydrogenation process at surface sites in which nitro activation is a limiting step.

**Figure 4:**
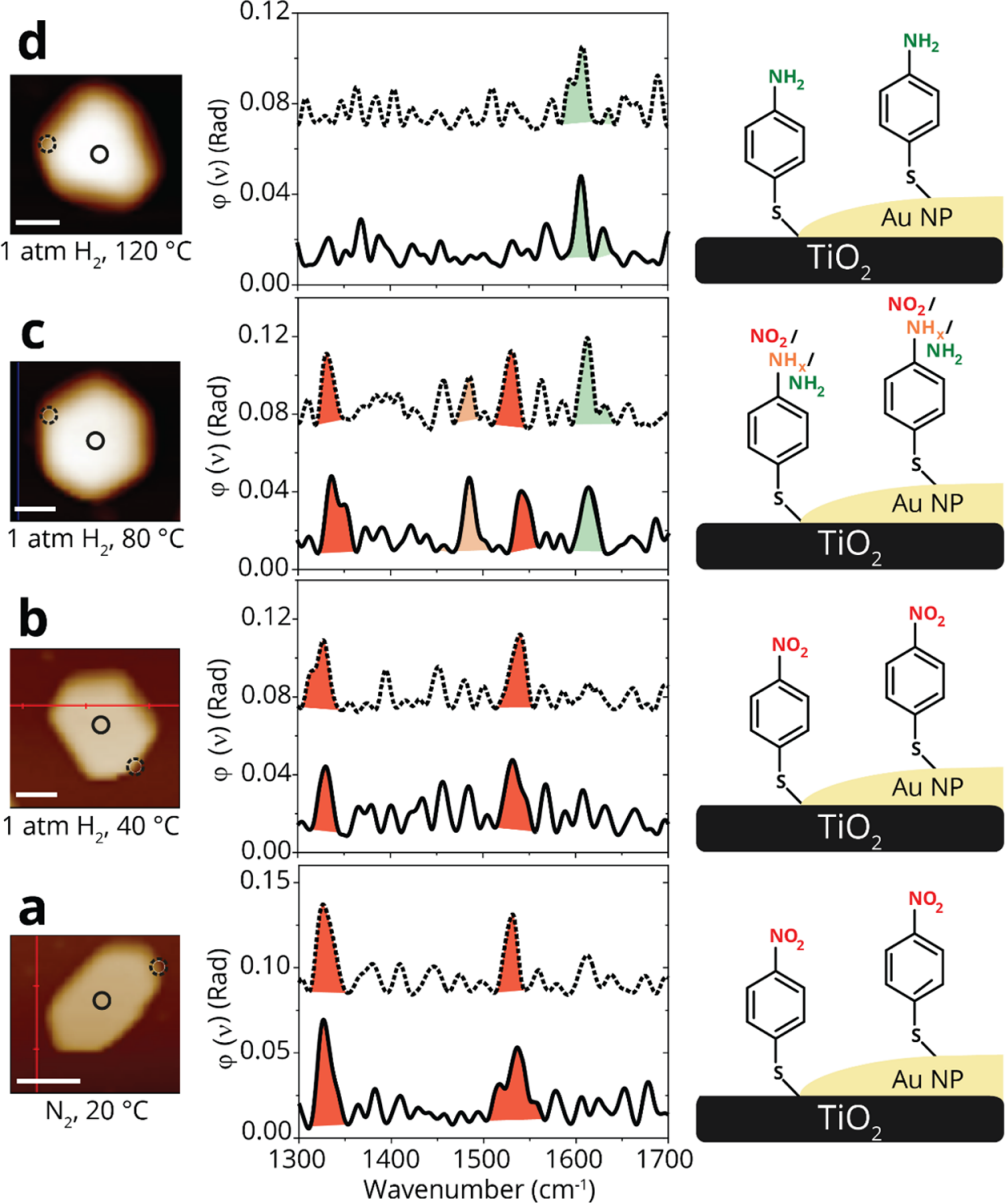
AFM images and IR nanospectroscopy measurements of TiO_2_-supported Au particles that were coated with *p*-NTP and exposed to various reducing conditions. The topography of Au particles was measured by AFM (left). IR spectrum was locally measured at the center and edge of the probed particle (middle). The location of the IR measurement is marked by solid and dashed circles in the AFM image, and the corresponding IR spectra are plotted in solid and dashed lines. Schematic description of the *p*-NTPs located on the center and edge of the Au particle, as identified by local IR measurements (right). AFM and IR nanospectroscopy measurements were conducted at rt (a) and after exposure of the sample to 1 atm H_2_ at 40 (b), 80 (c), and 120 °C (d). The IR peaks were colored for clarity. The nitro group was color-coded in red, and the amine group was color-coded in green, while intermediates such as hydroxylamine were colored in orange. Scale bar in all AFM images is 100 nm.

TiO_2_-supported Au particles were coated with *p*-NTP, and their site-dependent reactivity was monitored by IR nanospectroscopy measurements. The particles’ morphology was measured by AFM and followed by localized IR nanospectroscopy measurements at the edge and center of the Au particle (Figure 4a, left). Two peaks were detected in the IR spectra, centered at 1325 and 1530 cm^–1^ and correlated to the symmetric and asymmetric stretches of the nitro groups, respectively (Figure 4a, middle).

Exposure of the sample to mild reducing conditions (1 atm H_2_, 40 °C) did not yield a measurable change in the IR spectra (Figure 4b). Once the temperature was raised to 80 °C, two new peaks were detected at 1485 and 1615 cm^–1^ and attributed to hydroxylamine and amine, respectively (Figure 4c). Detection of the hydroxylamine signature implied that a partial reduction of the nitro groups occurred on the Au surface. When the sample was exposed to harsher reducing conditions (1 atm H_2_, 120 °C, 10 h) a peak was identified at 1615 cm^–1^, corresponding to the N–H bending (Figure 4d). This result indicates that a complete nitro-to-amine reduction was achieved for *p*-NTP on Au/TiO_2_ at 120 °C. It should be noted that nitro-to-amine reduction in NO_2_-NHCs was already detected at 40 °C while using TiO_2_-supported Au particles (Figure 2). Although *p*-NTP reduction occurred at a higher temperature than that of NO_2_-NHCs, site-independent reactivity was identified in both cases.

The influence of SiO_2_ and Al_2_O_3_ supports on the reactivity of Au particles toward nitro reduction in *p*-NTP was studied (Supporting Information Figures S9 and S10, respectively). The averaged site-dependent reactivity pattern, based on IR nanospectroscopy measurements of ∼10 particles, is schematically presented in Figure 3b. A complete nitro-to-amine reduction was identified at 120 °C for TiO_2_-supported Au particles. Lower reactivity was detected with SiO_2_, in which only partial reduction was obtained at 120 °C and no reduction was identified using Al_2_O_3_ as a support. In accordance with the results obtained for NO_2_-NHC, site-independent reactivity toward *p*-NTP reduction was identified for Au nanoparticles supported on the three different metal-oxides.

Comparison of nitro reduction of *p*-NTP versus NO_2_-NHCs revealed two main differences: (i) higher temperatures were required for *p*-NTP reduction than that of NO_2_-NHC reduction; (ii) intermediates were detected in *p*-NTP reduction and were not detected in NO_2_-NHC reduction. These differences were connected with the higher surface proximity and reducibility of the nitro groups in NO_2_-NHCs.^58,59^

The higher activation energy barrier for nitro reduction in *p*-NTP enabled the detection of intermediates on Au particles; however, even upon tuning the nitro reduction, homogeneous distribution of intermediates and reduction products were identified on the surface of the various particles. The site-independent reactivity pattern observed for *p*-NTP complements the results for NO_2_-NHCs, suggesting a general mechanism for the hydrogenation reaction on oxide-supported Au particles. In essence, Au surface sites are equal in their ability to activate the nitro groups toward reduction. Once the reaction conditions enable dissociative chemisorption of molecular hydrogen at the interface, highly efficient atomic hydrogen diffusion provides a supply of hydrogen atoms migrating hundreds of nanometers into the particle’s interior. The constant supply of active hydrogen atoms rendered these purportedly low catalytically active surface sites as reactive as interface sites toward hydrogenation.

Near-ambient pressure X-ray photoelectron spectroscopy (NAP-XPS) measurements were conducted to link the reactivity that was measured on single Au particles with that of a large ensemble of oxide-supported Au particles. N 1s NAP-XPS measurements probed the nitro reduction yield of oxide-supported Au particles that were coated with *p*-NTP and exposed to 0.1 Torr H_2_ at variable temperatures (Supporting Information Figures S11 and S12). The XPS results show the immanent influence of the oxide support on the reactivity of Au particles toward nitro reduction, with the oxide-dependent reactivity pattern of TiO_2_ > SiO_2_ > Al_2_O_3_. Thus, the ensemble-based reactivity measurements complemented the IR nanospectroscopy data and show a similar pattern of the oxide influence on the reactivity of supported Au particles.

**Figure 5:**
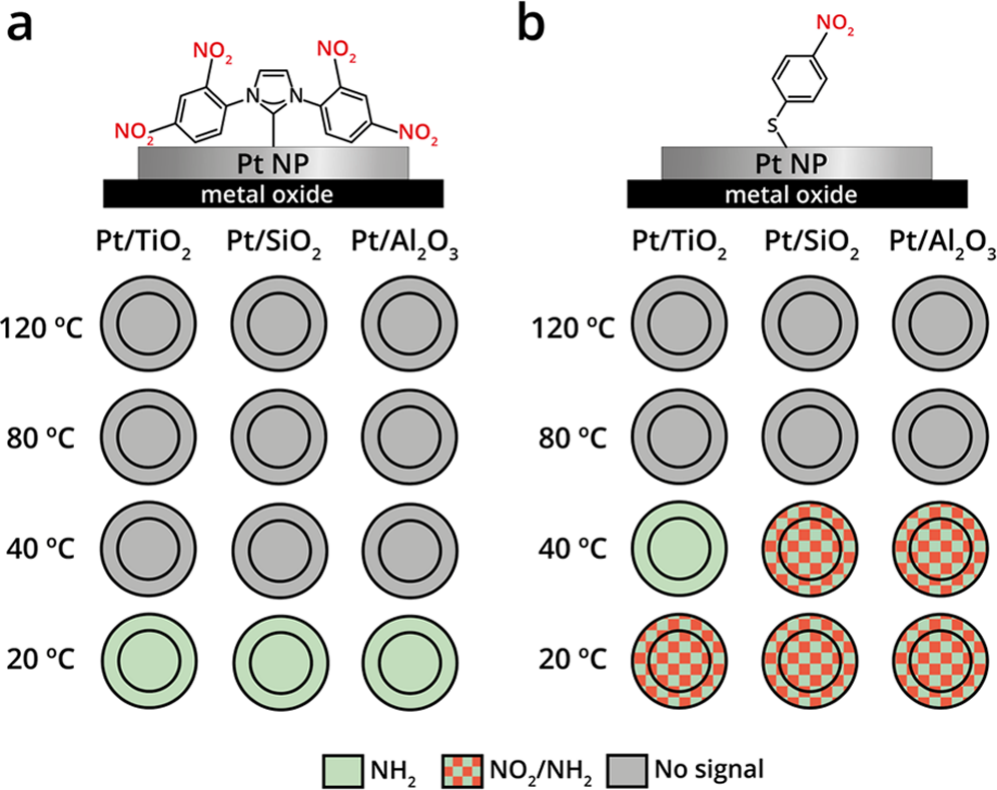
Schematic representation of the reactivity pattern toward nitro reduction of NO_2_-NHCs and *p*-NTP on oxide-supported Pt particles. The reactivity toward nitro reduction of (a) NO_2_-NHC- and (b) *p*-NTP-coated Pt particles that were deposited on different oxides was probed by IR nanospectroscopy measurements and schematically shown. The outer ring and the inner circle correspond to the reactivity that was detected at the oxide–metal interface and on the central part of the particles. The reactivity on the different sites was color-coded. The nitro group was color-coded in red, and the amine group was color-coded in green.

The central influence of the Au-oxide interface in facilitating hydrogenation reactions across the Au particle results from the deteriorated reactivity of Au metal sites that are located further away from the interface; therefore, it was posited that the oxide–metal interface will be less influential once Au will be replaced with a more reactive metal, such as Pt. To test this hypothesis, Pt particles were prepared on TiO_2_, SiO_2_, and Al_2_O_3_ supports and coated with NO_2_-NHCs and *p*-NTP. Subsequently, their reactivity toward nitro reduction was mapped following exposure to various reducing conditions. Single-particle IR nanospectroscopy data is shown in the Supporting Information (Figures S13 and S14) and the spectroscopic conclusions are schematically presented in Figure 5.

Nitro-to-amine reduction was identified across the surface of NO_2_-NHCs coated Pt particles already at room temperature, with no influence of the oxide support on the reactivity. Exposure of the samples to harsher reducing conditions (*T* > 40 °C) led to disappearance of the IR signal (Figure 5a), correlated to decomposition of the NHCs.^58,59^ The high reactivity of Pt surface sites dominated the nitro reduction reaction; therefore, the influence of the oxide support on reactivity was not observed. These results show that, within the measured temperature range, sites located at the oxide–metal interface do not noticeably differ in their reactivity compared to sites located in the interior of the Pt particle.

Interestingly, IR nanospectroscopy measurements previously identified higher reactivity at the perimeter of single Pt particles in oxidation reactions.^13,43^ The dissimilarity between site-dependent reactivity in oxidation reactions and site-independent reactivity in hydrogenation reactions on Pt nanoparticles can be connected to variations in the dissociative chemisorption yield and surface-diffusion coefficient of oxygen and hydrogen.^65−67^ The lower activation energy barrier for dissociative chemisorption of hydrogen, in comparison to that of oxygen, and its higher surface diffusion rates led to site-independent reactivity in hydrogenation reactions.

The reactivity of oxide-supported Pt particles toward nitro reduction of *p*-NTP was measured (Supporting Information Figure S14) and the averaged reactivity patterns are presented in Figure 5b. Partial nitro reduction was already detectable at room temperature. Exposure of the samples to mild reducing conditions (1 atm H_2_, 40 °C, 10 h) did not alter the reactivity of SiO_2_- and Al_2_O_3_-supported Pt particles; however, a complete reduction was observed for TiO_2_-supported Pt particles (Figure 5b). Exposure of the samples to higher reduction temperatures eliminated the IR signals, which was correlated to decomposition of *p*-NTP on the Pt surface. Deteriorated nitro reduction was detected when *p*-NTP was used, compares to NO_2_-NHCs, on Pt nanoparticles.

The hydrogenation reactivity pattern toward *p*-NTP reduction on oxide-supported Au and Pt particles led to the following conclusions: (i) higher reactivity was detected when Au and Pt particles were supported on TiO_2_; (ii) Pt particles demonstrated higher reactivity than Au particles, and their reactivity was only mildly influenced by the oxide support; and (iii) hydroxylamine intermediates were detected on Au particles and were not detected on Pt particles during *p*-NTP reduction.

Integration of the experimental data revealed site-independent reactivity toward nitro reduction for both Au and Pt particles, while using either NO_2_-NHC or *p*-NTP as indicators. However, the origins of this reactivity pattern were different for the two metals. For Au nanoparticles, dominant influence of the oxide support on the reactivity was detected with TiO_2_ > SiO_2_ > Al_2_O_3_ in activating the hydrogenation reaction. The reactivity pattern of Au particles, which, on the one hand, shows site-independent reactivity and, on the other hand, shows high influence of the oxide support, can be rationalized by highly efficient intraparticle diffusion of hydrogen atoms from the metal–oxide interface to the interior part of the particle. These results demonstrate the active nature of Au metal sites and their capability to strongly interact with the nitro groups to enable their reduction. The oxide support had only a minor influence on the reactivity of Pt particles toward nitro reduction. Highly reactive Pt sites facilitated the dissociation of H_2_ and strongly interact with the nitro groups to enable their hydrogenation, leading to high reactivity which is negligibly influenced by the oxide support.

Previous studies have identified that optimal reactivity in both hydrogenation and oxidation reactions is achieved with oxide-supported Au nanoparticles in the size range of 1–5 nm.^57,68,69^ Based on these studies and others, it was concluded that reactivity is enhanced at the Au/oxide interface and that small nanoparticles (<5 nm), in which the interface to bulk ratio is maximized, are essential for optimized catalytic reactivity. However, single-particle reactivity measurements, showed herein, did not reveal site-dependent variations in the reactivity pattern and even when intermediates were detected on the particles, they were detected both at the interface and the interior part of the particle. In oxidation reactions, on the other hand, both site-dependent reactivity and selectivity were detected on single particles using IR nanospectroscopy.^43,45^

The obtained results demonstrate similar affinity toward nitro reduction on sites that are located at the interface and sites located at the interior part of Au particles. These observations imply that the bottleneck for reactivity induction is the ability to supply atomic hydrogen from the interface to the inner part of the Au particle. Moreover, they support the hypothesis that high reactivity toward hydrogenation reactions can be also achieved on large (>5 nm) Au nanoparticles once sufficient supply of hydrogen atoms is provided throughout the metal surface.

## Conclusions

Site-independent reactivity was identified in nitro reduction on oxide-supported Au and Pt particles by conducting IR nanospectroscopy measurements on single particles. Comparative analysis revealed that TiO_2_ > SiO_2_ > Al_2_O_3_ in facilitating the reactivity of oxide-supported Au particles, while the oxide support had only a minor influence on the reactivity of Pt particles. Single-particle reactivity measurements revealed that hydrogen dissociation at the oxide–metal interface was a dominant factor in nitro reduction on Au particles. This step was followed by efficient diffusion of hydrogen atoms from the interface that enabled nitro reduction throughout the Au surface. These results demonstrate that Au metal sites can strongly interact with nitro groups to facilitate their activation, but cannot efficiently dissociate molecular hydrogen. On Pt particles, on the other hand, both dissociative chemisorption of hydrogen and activation of nitro groups were activated by Pt metal sites, with no reactivity enhancement at interface sites. While this study does not provide quantitative measurements of rate data, it provides trends that result in useful insights into relative reactivity pattern of oxide-supported Au nanoparticles. The molecular-level understanding of hydrogenation reaction mechanism on metallic nanoparticles, that was identified herein, demonstrates that the reactivity of Au metal sites is sufficient for activation of unsaturated groups toward hydrogenation. Thus, the requirement for small oxide-supported Au nanoparticles for the activation of hydrogenation reaction can be eased by efficient diffusion of hydrogen atoms from the interface to the interior part of the particle.

## Supporting Information Available

High-resolution scanning electron microscopy (HR-SEM), XRD, and XPS measurements of oxide-supported Au particles; IR nanospectroscopy measurements of oxide-supported Au and Pt particles; and NAP-XPS measurements (PDF)
