# Supplementary material for: Site-Independent Hydrogenation Reactions on Oxide-Supported Au Nanoparticles Facilitated by Intraparticle Hydrogen Atom Diffusion
Source: ACS Catal. 2021 Jul 21;11(15):9875–84. doi: 10.1021/acscatal.1c01987 (PMC9223368; doi:10.1021/acscatal.1c01987)
Supplement: Supplementary file 1 — cs1c01987_si_001.pdf [file cs1c01987_si_001.pdf]

Supporting Information

Site-Independent Hydrogenation Reactions on Oxide-Supported Au Nanoparticles Facilitated by Intra-particle Hydrogen Atom Diffusion

Shahar Dery[1,2], Hillel Mehlman[1,2], Lillian Hale[3], Mazal Carmiel-Kostan[1,2], Reut Yemini[4,5], Tzipora Ben-Tzvi[1,2], Malachi Noked[4,5], F. Dean Toste[3] and Elad Gross[1,2] *

1. Institute of Chemistry, The Hebrew University, Jerusalem 91904, Israel
2. The Center for Nanoscience and Nanotechnology, The Hebrew University, Jerusalem 91904, Israel
3. Department of Chemistry, University of California, Berkeley, California 94720, USA
4. Department of Chemistry, Bar Ilan University, Ramat Gan, 5290002, Israel
5. Bar-Ilan Institute of Nanotechnology and Advanced Materials, Ramat Gan, 5290002, Israel

Corresponding author

* Elad Gross: elad.gross@mail.huji.ac.il

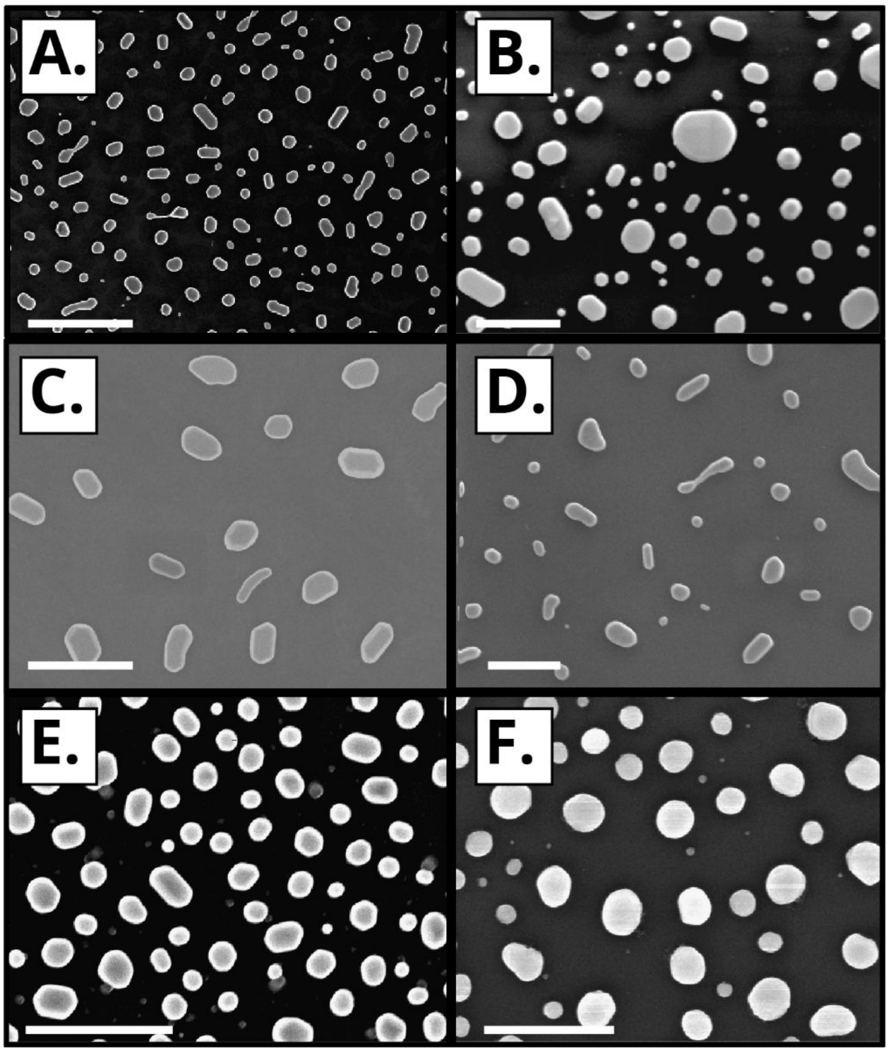

Figure S1. HR-SEM images of Au and Pt particles that were deposited on metal oxides. (**a**) Au/TiO_2; (**b**) Pt/TiO_2; (**c**) Au/SiO_2; (**d**) Pt/SiO_2; (**e**)Au/Al_2O_3; (**f**) Pt/Al_2O_3. Scale bar in all images is 400 nm.

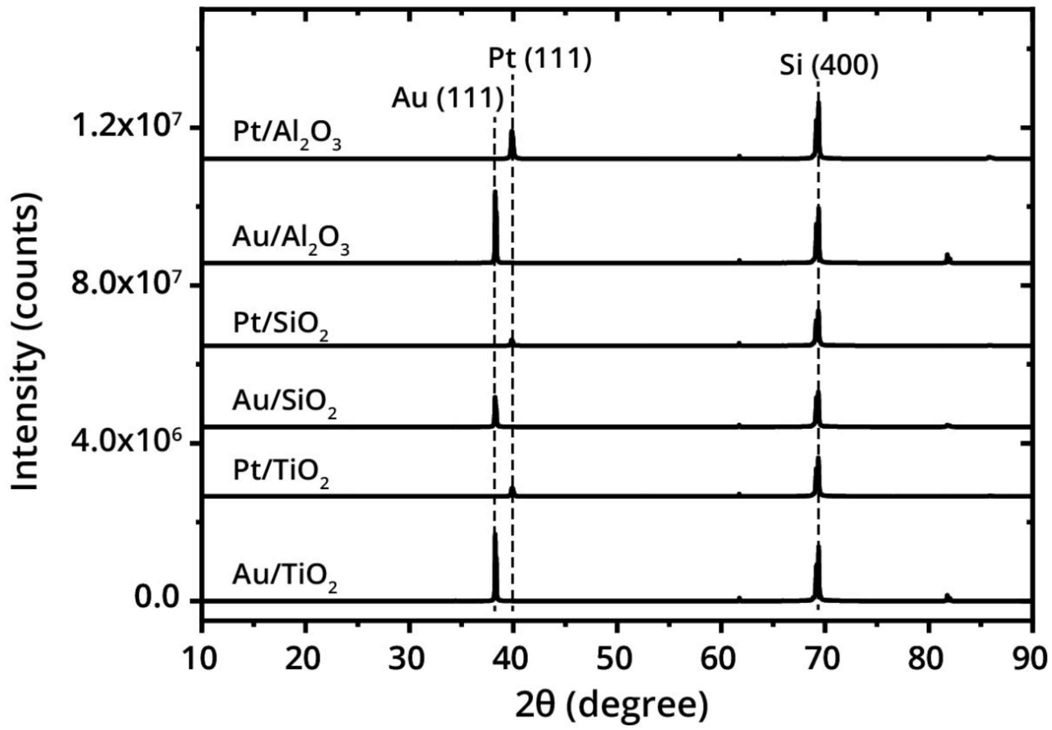

Figure S2. XRD spectra of Au and Pt particles that were deposited on various metal oxides and coated with NO_2-NHCs.

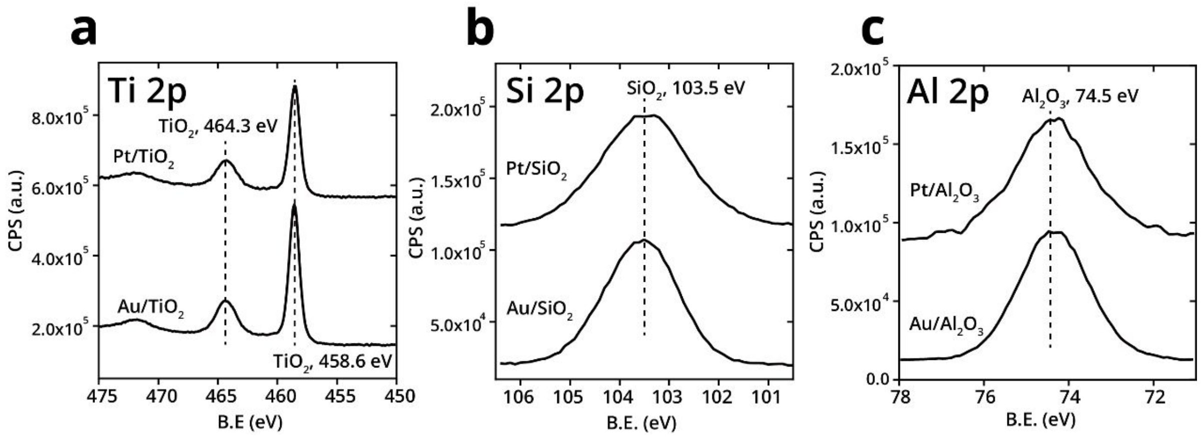

Figure S3. XPS spectra of Ti 2p (a) Si 2p (b) and Al 2p (c) of Au and Pt NPs deposited on TiO_2 (a), SiO_2 (b) and Al_2O_3 (c).

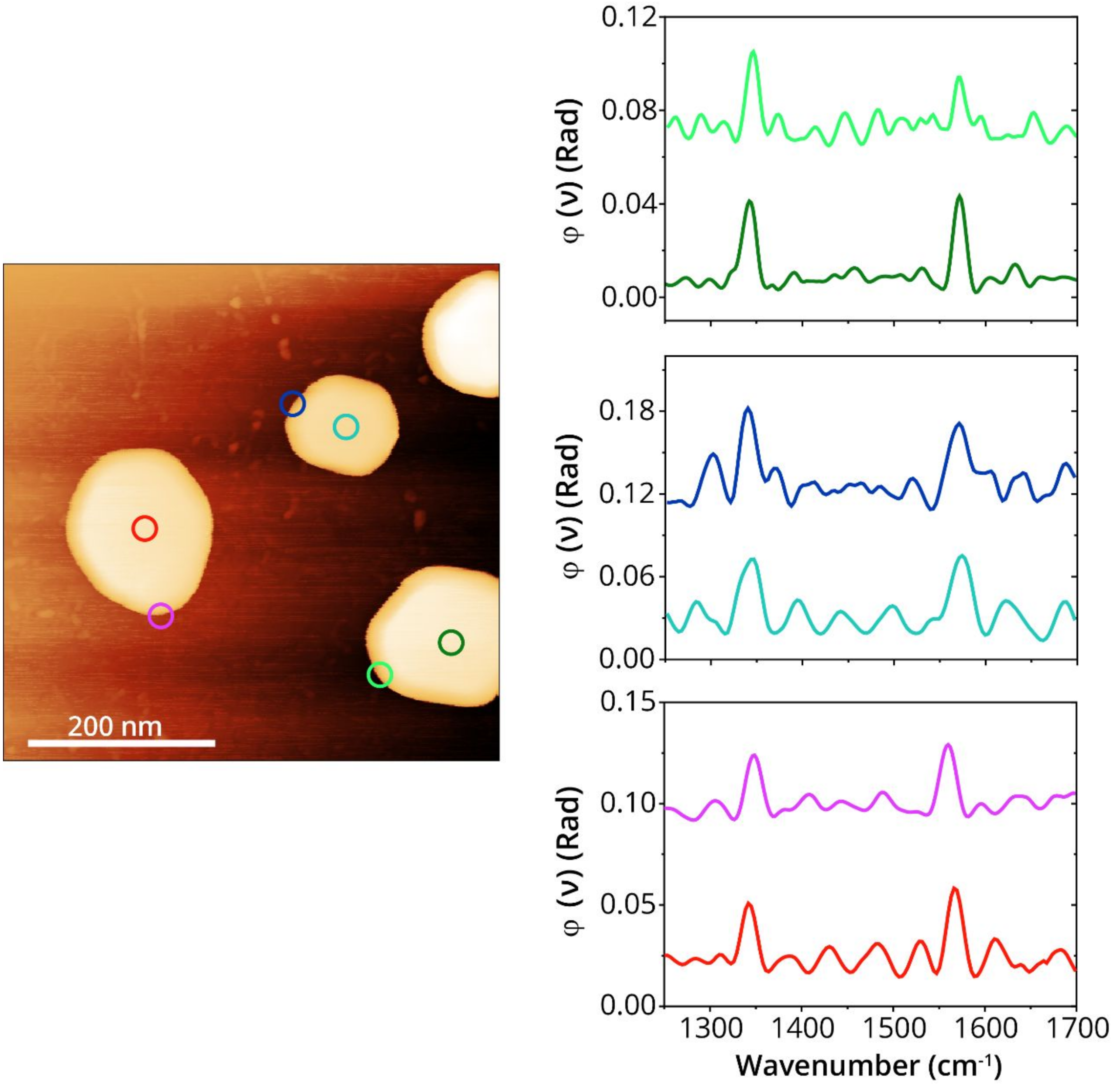

Figure S4. AFM image and IR nanospectroscopy measurements of Au particles that were deposited on TiO_2 and coated with NO_2-NHCs. The topography of Au particles was measured by AFM (left). IR spectra were locally measured at the center and edge of the probed particles (right panel). The location of each IR measurement is marked by colored circles in the AFM image and the corresponding IR spectra are plotted with the same color coding in the right panel. All measurements were conducted at room temperature and prior to exposure to reducing conditions.

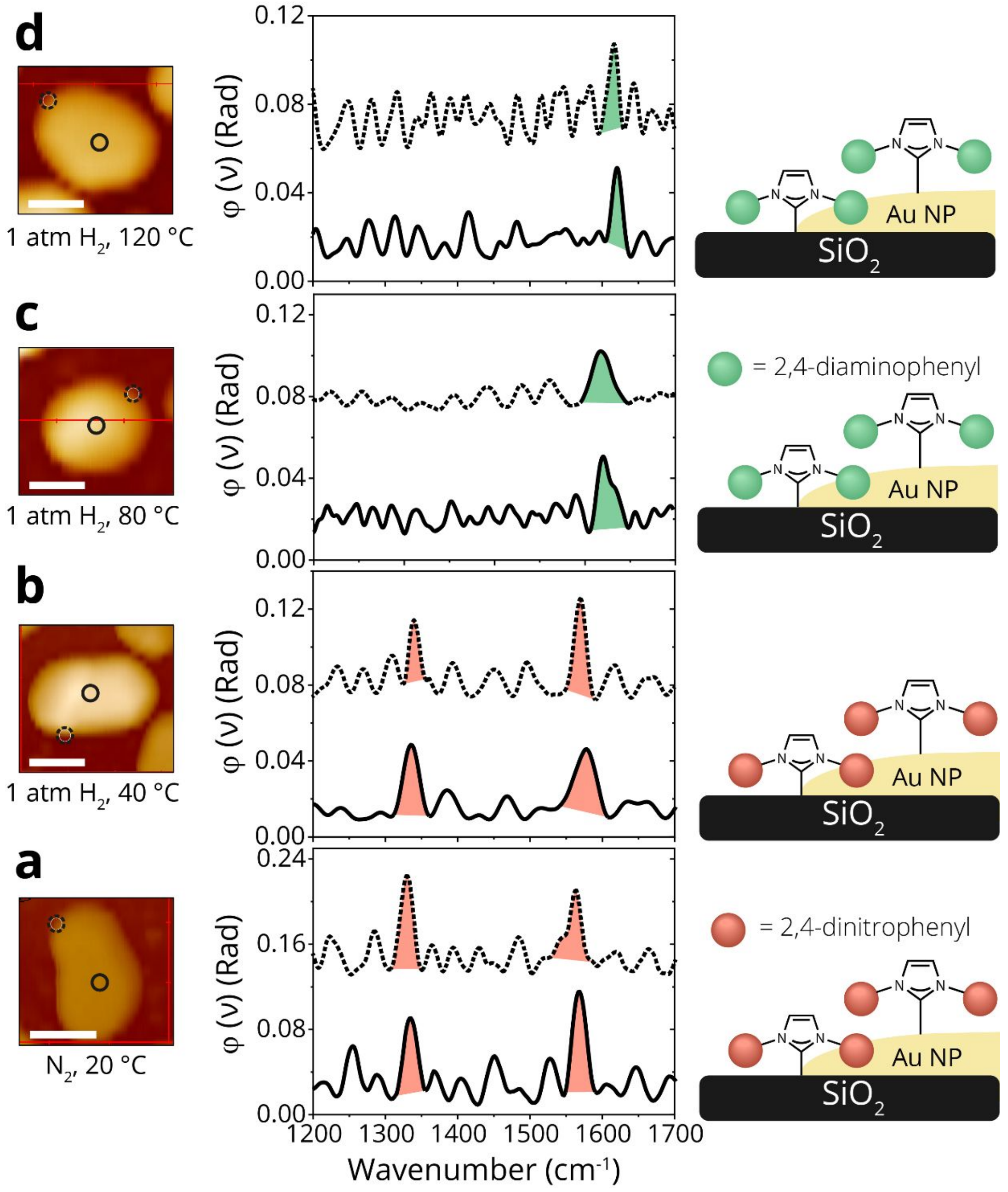

Figure S5. AFM images and IR nanospectroscopy measurements of Au particles that were deposited on SiO_2 and coated with NO_2-NHCs. The topography of Au particles was measured by AFM (left panels). IR spectrum was locally measured at the center and edge of the probed particle (central panels). The location of the IR measurement is marked by solid and dashed circles in the AFM image and the corresponding IR spectra are plotted in solid and dashed lines, respectively. Schematic description of the NHCs located on the center and edge of the Au particle, as identified by local IR measurements, are shown in the right panels. AFM and IR nanospectroscopy measurements were conducted at rt **(a)** and after exposure of the sample to 1 atm H_2 at 40 **(b)**, 80 **(c)** and 120 °C **(d).** Scale bar in all AFM images is 100 nm.

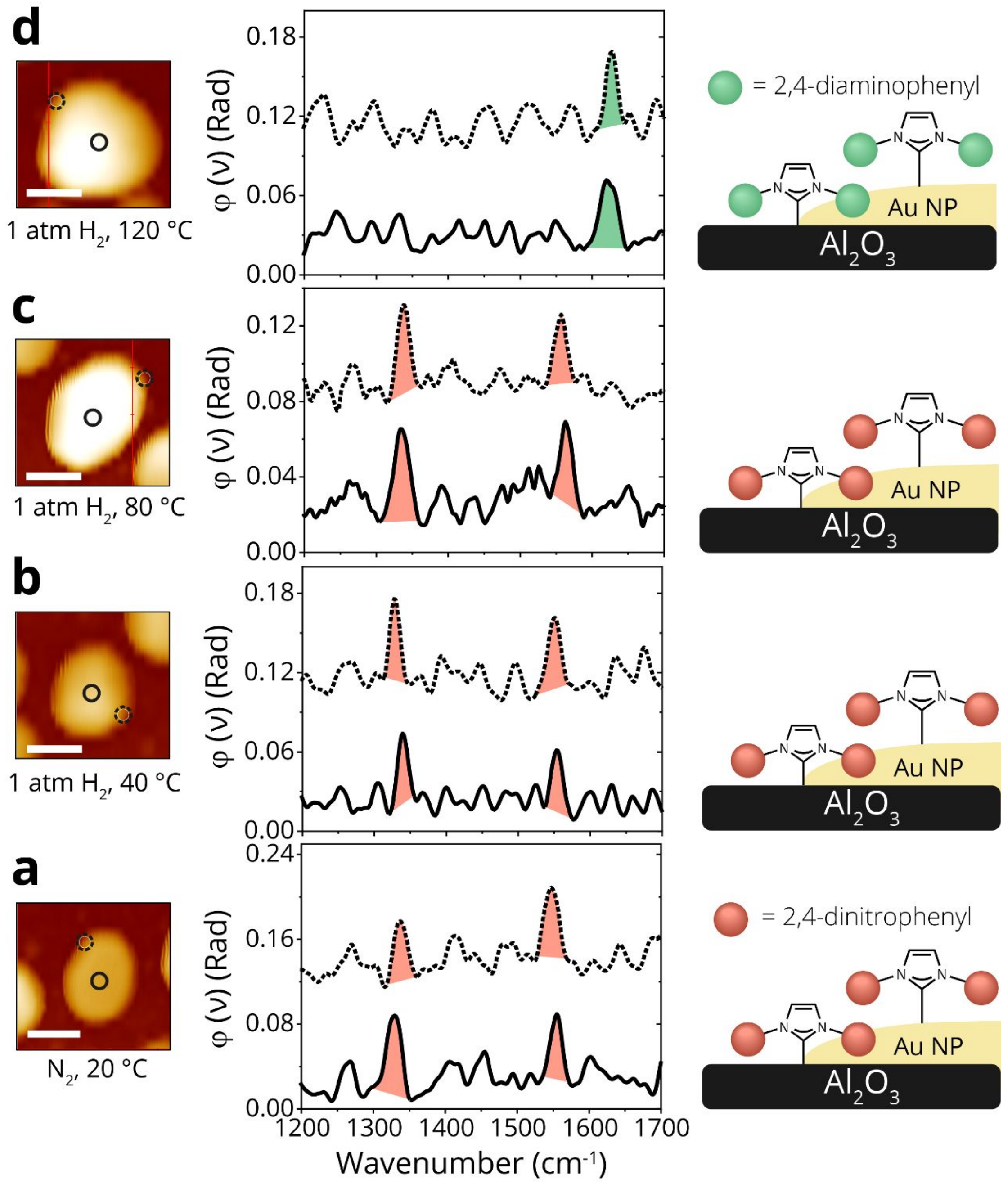

Figure S6. AFM images and IR nanospectroscopy measurements of Au particles that were deposited on Al_2O_3 and coated with NO_2-NHCs. The topography of Au particles was measured by AFM (left panels). IR spectrum was locally measured at the center and edge of the probed particle (central panels). The location of the IR measurement is marked by solid and dashed circles in the AFM image and the corresponding IR spectra are plotted in solid and dashed lines, respectively. Schematic description of the NHCs located on the center and edge of the Au particle, as identified by local IR measurements, are shown in the right panels. AFM and IR nanospectroscopy measurements were conducted at rt **(a)** and after exposure of the sample to 1 atm H_2 at 40 **(b)**, 80 **(c)** and 120 °C **(d).** Scale bar in all AFM images is 100 nm.

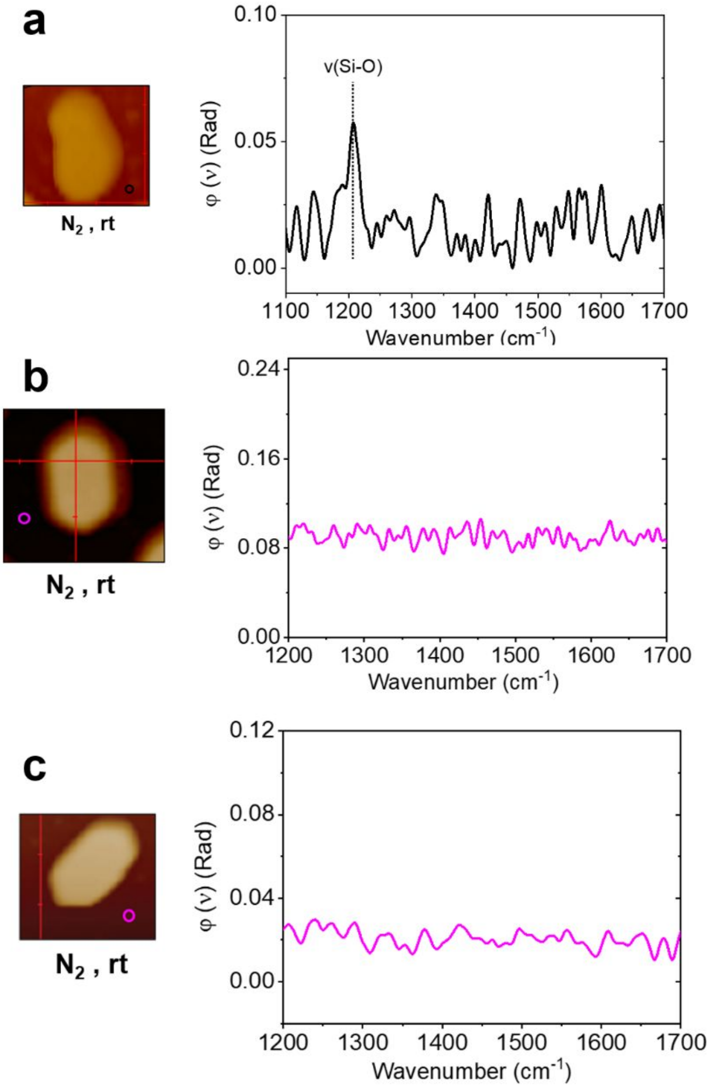

Figure S7. AFM images and IR nanospectroscopy measurements were performed on SiO_2 (a) Al_2O_3 (b) and TiO_2 (c) films on which Au nanoparticles were prepared and coated with NO_2-NHCs.

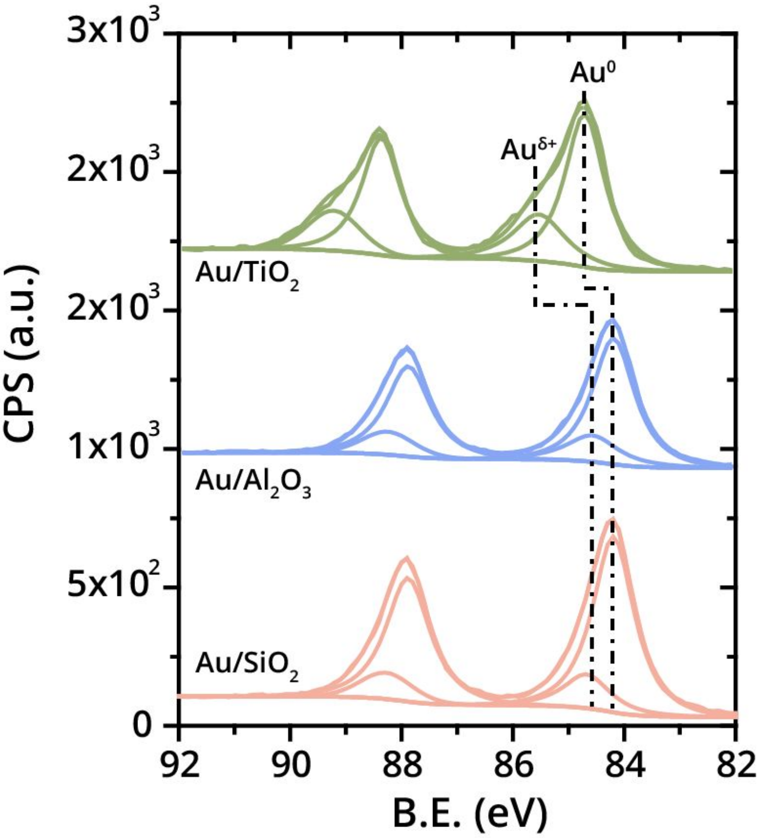

Figure S8. Au4f XPS spectrum of Au particles that were deposited on SiO_2, Al_2O_3 and TiO_2 (pink, blue and green colored spectra, respectively) and coated with p-NTP.

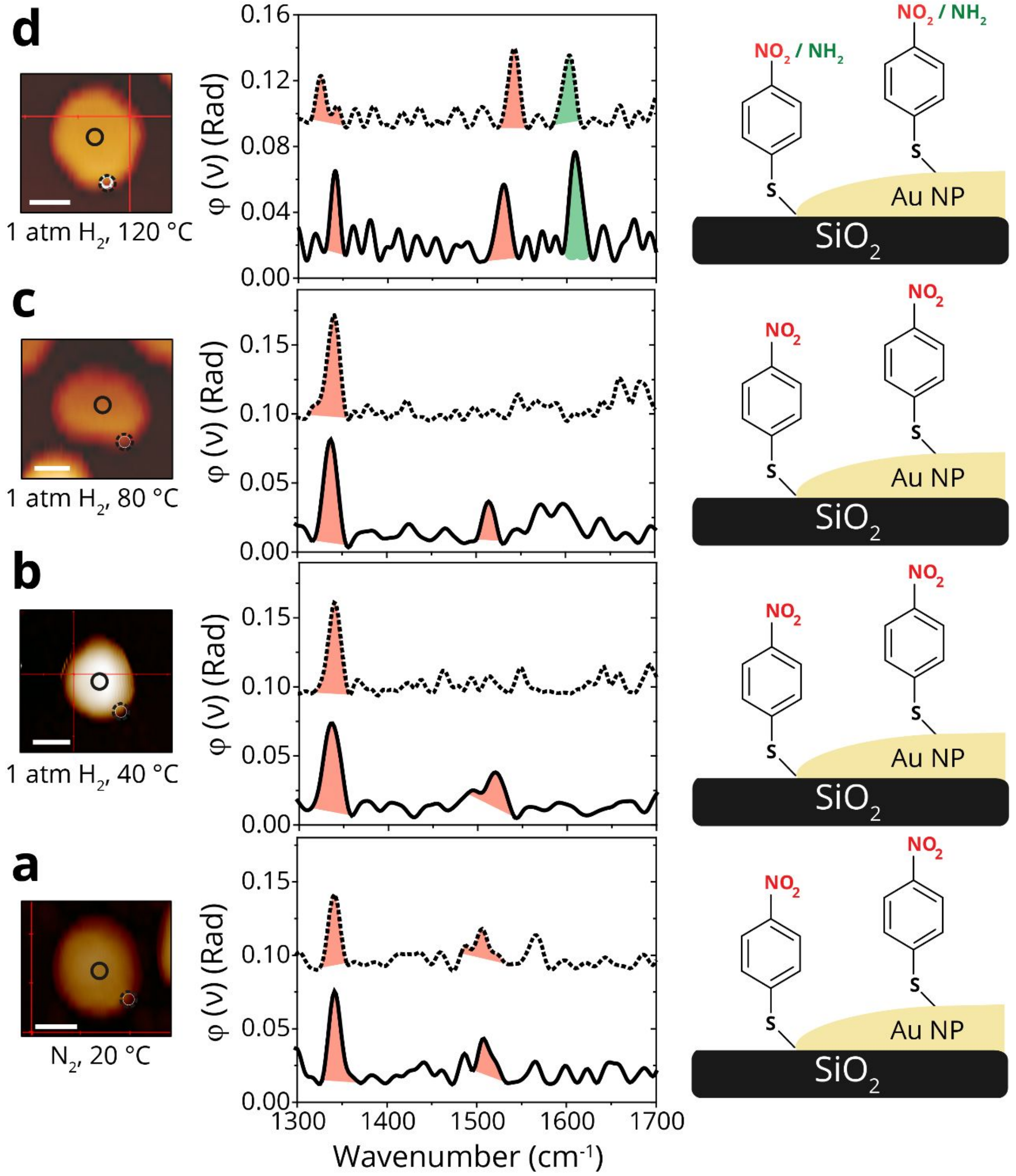

Figure S9. AFM images and IR nanospectroscopy measurements of Au particles that were deposited on SiO_2 and coated with p-NTP. The topography of Au particles was measured by AFM (left panels). IR spectrum was locally measured at the center and edge of the probed particle (central panels). The location of the IR measurement is marked by solid and dashed circles in the AFM image and the corresponding IR spectra are plotted in solid and dashed lines, respectively. Schematic description of the p-NTPs located on the center and edge of the Au particle, as identified by local IR measurements, are shown in the right panels. AFM and IR nanospectroscopy measurements were conducted at rt **(a)** and after exposure of the sample to 1 atm H_2 at 40 **(b)**, 80 **(c)** and 120 °C **(d).** Scale bar in all AFM images is 100 nm.

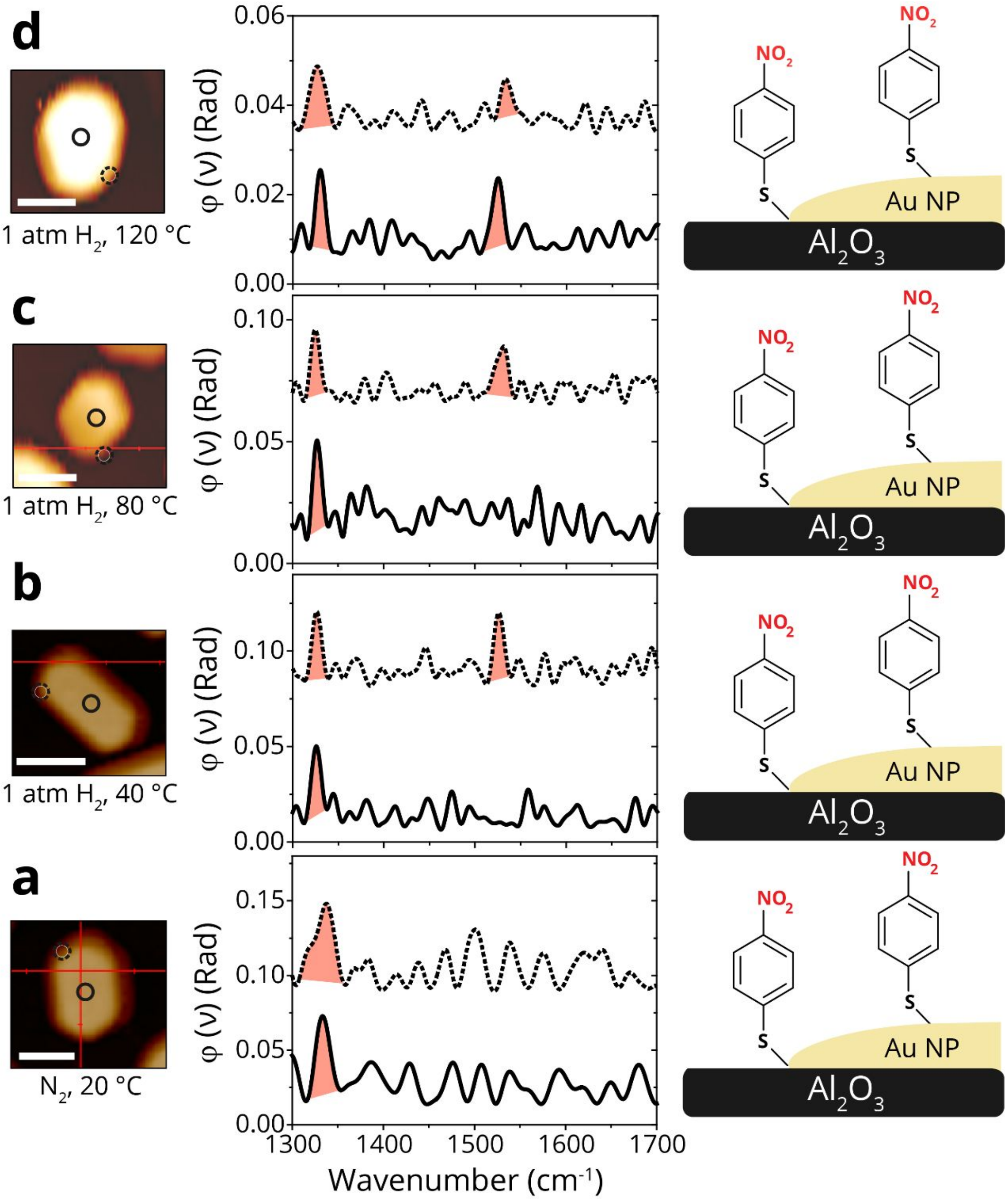

Figure S10. AFM images and IR nanospectroscopy measurements of Au particles that were deposited on Al_2O_3 and coated with p-NTP. The topography of Au particles was measured by AFM (left panels). IR spectrum was locally measured at the center and edge of the probed particle (central panels). The location of the IR measurement is marked by solid and dashed circles in the AFM image and the corresponding IR spectra are plotted in solid and dashed lines, respectively. Schematic description of the p-NTPs located on the center and edge of the Au particle, as identified by local IR measurements, are shown in the right panels. AFM and IR nanospectroscopy measurements were conducted at rt **(a)** and after exposure of the sample to 1 atm H_2 at 40 **(b)**, 80 **(c)** and 120 °C **(d).** Scale bar in all AFM images is 100 nm.

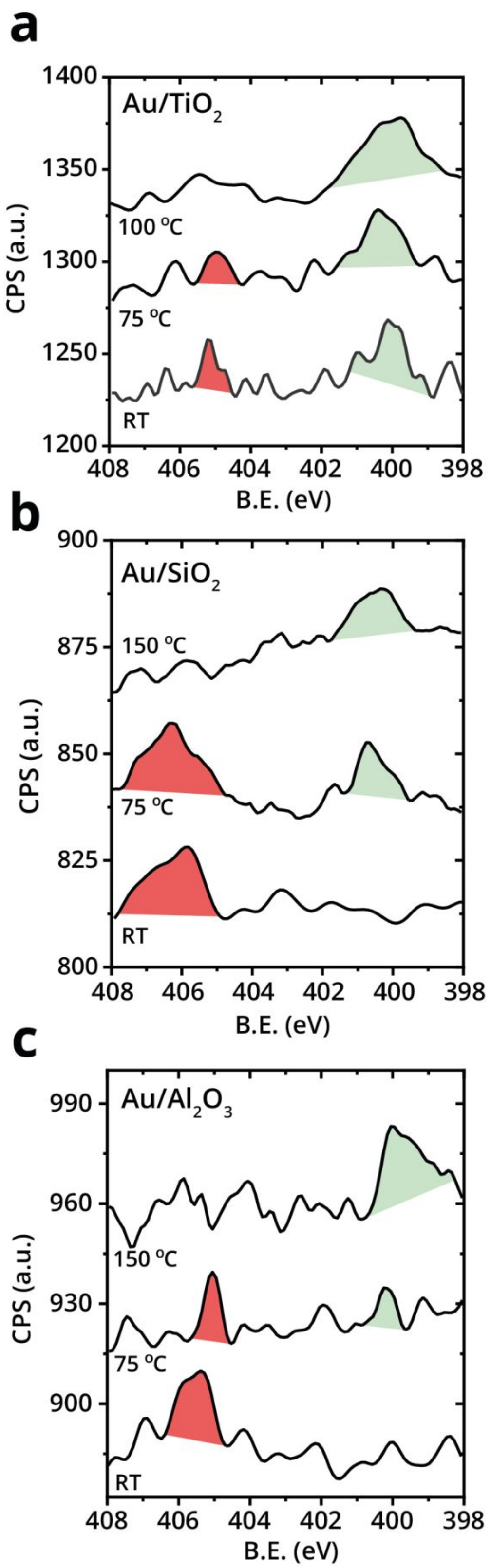

Figure S11. N1s NAP-XPS spectra of p-NTP coated Au particles that were deposited on TiO_2 (a), SiO_2 (b) and Al_2O_3 (c) and exposed to 0.1 Torr H_2 at various surface temperatures.

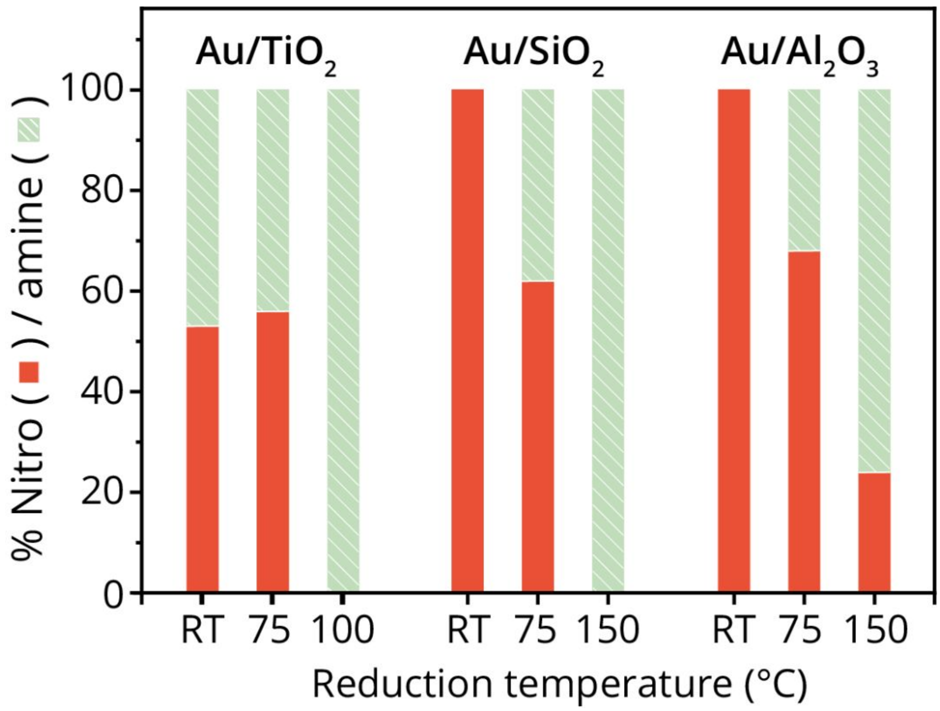

Figure S12. XPS-based analysis of nitro reduction yield in p-NTP coated Au particles. The nitro reduction yield of p-NTPs on Au particles that were deposited on various oxides was quantified by analysis of the N1s NAP-XPS spectra. NAP-XPS measurements were conducted at 0.1 Torr H_2 and variable temperature.

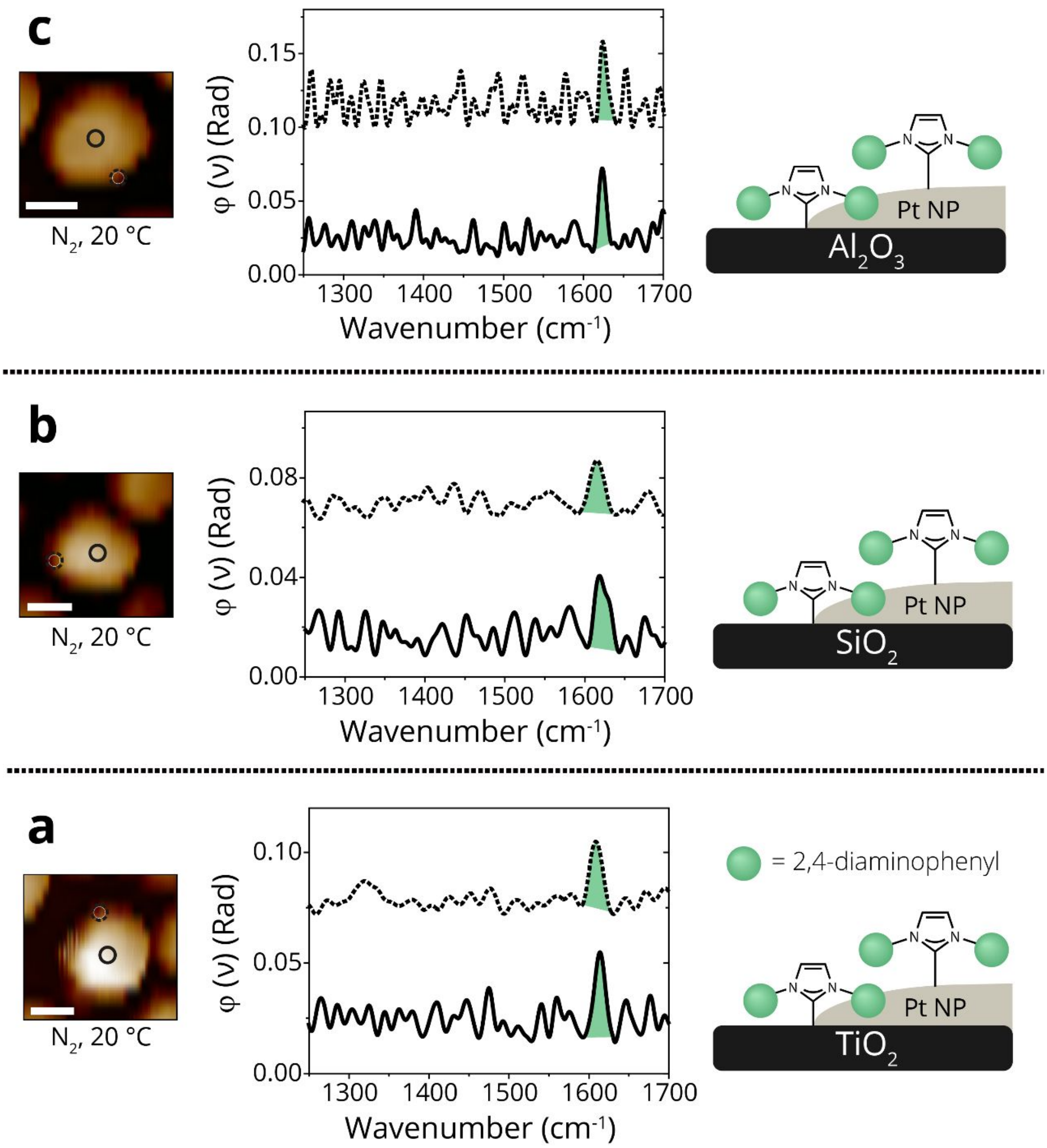

Figure S13. AFM images and IR nanospectroscopy measurements of Pt particles that were deposited on TiO_2 (a), SiO_2 (b) and Al_2O_3 (c) and coated with NO_2-NHCs. The topography of Pt particles was measured by AFM (left panels). IR spectrum was locally measured at the center and edge of the probed particle (central panels). The location of the IR measurement is marked by solid and dashed circles in the AFM image and the corresponding IR spectra are plotted in solid and dashed lines, respectively. Schematic description of the NHCs located on the center and edge of the Pt particle, as identified by local IR measurements, are shown in the right panels. AFM and IR nanospectroscopy measurements were conducted at rt. Scale bar in all AFM images is 100 nm.

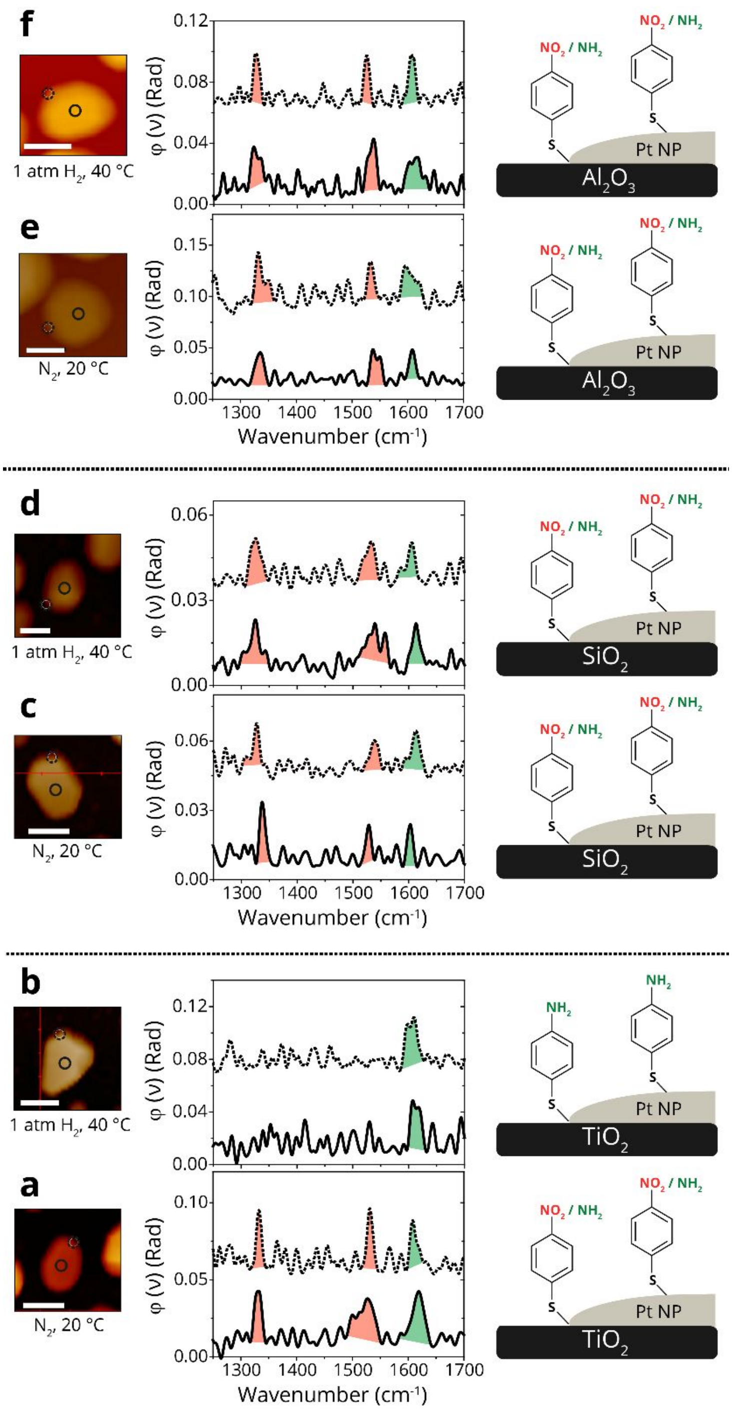

Figure S14. AFM images and IR nanospectroscopy measurements of Pt particles that were deposited on TiO_2 (a-b), SiO_2 (c-d) and Al_2O_3 (e-f) and coated with p-NTP. The topography of Pt particles was measured by AFM (left panels). IR spectrum was locally measured at the center and edge of the probed particle (central panels). The location of the IR measurement is marked by solid and dashed circles in the AFM image and the corresponding IR spectra are plotted in solid and dashed lines, respectively. Schematic description of the NHCs located on the center and edge of the Pt particle, as identified by local IR measurements, are shown in the right panels. AFM and IR nanospectroscopy measurements were conducted at rt (a, c and e) and after exposure to 1 atm H_2 at 40°C (b, d and f). Scale bar in all AFM images is 100 nm.
